# Co-delivery of a laminin-111 supplemented hyaluronic acid based hydrogel with minced muscle graft in the treatment of volumetric muscle loss injury

**DOI:** 10.1371/journal.pone.0191245

**Published:** 2018-01-12

**Authors:** Stephen M. Goldman, Beth E. P. Henderson, Thomas J. Walters, Benjamin T. Corona

**Affiliations:** United States Army Institute of Surgical Research, JBSA Fort Sam Houston, Texas, United States of America; University of Kansas Medical Center, UNITED STATES

## Abstract

Minced muscle autografting mediates *de novo* myofiber regeneration and promotes partial recovery of neuromuscular strength after volumetric muscle loss injury (VML). A major limitation of this approach is the availability of sufficient donor tissue for the treatment of relatively large VMLs without inducing donor site morbidity. This study evaluated a laminin-111 supplemented hyaluronic acid based hydrogel (HA+LMN) as a putative myoconductive scaffolding to be co-delivered with minced muscle grafts. In a rat tibialis anterior muscle VML model, delivery of a reduced dose of minced muscle graft (50% of VML defect) within HA+LMN resulted in a 42% improvement of peak tetanic torque production over unrepaired VML affected limbs. However, the improvement in strength was not improved compared to a 50% minced graft-only control group. Moreover, histological analysis revealed that the improvement in *in vivo* functional capacity mediated by minced grafts in HA+LMN was not accompanied by a particularly robust graft mediated regenerative response as determined through donor cell tracking of the GFP^+^ grafting material. Characterization of the spatial distribution and density of macrophage and satellite cell populations indicated that the combination therapy damps the heightened macrophage response while re-establishing satellite content 14 days after VML to a level consistent with an endogenously healing ischemia-reperfusion induced muscle injury. Moreover, regional analysis revealed that the combination therapy increased satellite cell density mostly in the remaining musculature, as opposed to the defect area. Based on the results, the following salient conclusions were drawn: 1) functional recovery mediated by the combination therapy is likely due to a superposition of *de novo* muscle fiber regeneration and augmented repair of muscle fibers within the remaining musculature, and 2) The capacity for VML therapies to augment regeneration and repair within the remaining musculature may have significant clinical impact and warrants further exploration.

## Introduction

Skeletal muscle possesses a remarkable endogenous capacity for regeneration under cases of mild chemical or physical insult [1–5]. This regenerative capacity, however, is overwhelmed in cases of traumatic volumetric muscle loss (VML), wherein the wound healing response shifts towards a fibrotic repair paradigm which leads to persistent muscle weakness and long term disability [6]. Ideally, orthopaedic surgical care of complex musculoskeletal injuries presenting VML would include a regenerative therapy that can promote restoration of the lost contractile tissue and aid in restoring limb function. Unfortunately, there is currently no regenerative standard of care for VML injury. One developmental treatment paradigm is the transplantation of autologous muscle grafts in minced form [7–9]. Autologous minced muscle grafts (MG) are effective in promoting *de novo* muscle fiber regeneration and partial functional recovery in small and large animal models of VML [7, 9–11]. A major limitation of this approach is the availability of autologous tissue sources and the donor site morbidity associated with the procurement of the requisite tissue volume for a 100% volumetric repair of relatively large VML defects in large muscle units, such as the quadriceps muscles.

A potential enabling technology for minced grafts towards the repair of large VML defects is the co-delivery of myoconductive biomaterials [8, 12, 13]. That is, a scaffolding material that provides volumetric augmentation in a manner permissive to autologous minced graft-mediated functional regeneration. Autologous minced muscle grafts have been shown to promote skeletal muscle regeneration through graft-derived muscle progenitor cell activity[14–16], which is influenced by host immune cell infiltration in a manner akin to regeneration following recoverable muscle injuries, such as ischemia-reperfusion injury [1]. Notably, VML injury induces a heightened and prolonged inflammatory response [17–19] that is not conducive to skeletal muscle regeneration [20]. Therefore, it is reasonable that biomaterials that provide cues known to positively influence satellite cell function while damping the local immune response may perform in a myoconductive manner when co-delivered with minced muscle grafts.

The candidate scaffolding material evaluated herein is a semi-synthetic hydrogel composed of hyaluronic acid and laminin-111. The hyaluronic acid based hydrogel was chosen because of its unique anti-adhesive and anti-inflammatory properties [21, 22], which were postulated to effectively shield co-delivered minced grafts from the exacerbated immune response after VML injury. While the dearth of cell attachment sites on the hyaluronic acid molecule [23] imparts its properties as a migration barrier, it also offers a design space for the strategic addition of extracellular matrix (ECM) components to promote and control cell adhesion, migration, and proliferation in a selective manner [24]. As such, we incorporated laminin-111 as a component of the scaffolding system to promote myogenic cell fate processes. The laminin-111 isoform is one of the key ECM components present during the embryological development of skeletal muscle that has been shown to facilitate satellite cell migration and proliferation and improve skeletal muscle regeneration following injury [25–27]. Based on these properties, partial volumetric repair of autologous minced muscle grafts suspended in laminin-111 supplemented hyaluronic acid based hydrogels were evaluated in an established rodent model of VML injury.

## Materials & methods

### Experimental design

Male Lewis rats were divided into five experimental groups (n = 6–8 per group, endpoint) that each had VML injury in the middle third of the left TA muscle: (1) no repair, (2) repair with the base hydrogel alone (i.e. HA group), (3) repair with the laminin-111 supplemented hydrogel (i.e. HA+LMN), (4) repair with MG at a dosage of 50% of the defect mass encapsulated in the base hydrogel (i.e. 50% MG + HA), and (5) repair with MG at a dosage of 50% of the defect mass encapsulated in the laminin-111 supplemented hydrogel (i.e. 50% MG + HA+LMN). Rats survived to 2- and 8-weeks post injury before the muscles were harvested for histological and molecular analysis. Prior to harvest at the 8-week time-point, isometric tetanic strength of the injured and contralateral uninjured TA muscles was assessed *in vivo* via neural stimulation. An additional group of rats, underwent ischemia-reperfusion (I-R) injury (n = 16) and were survived to 14 and 28 days post-injury. Functional assessments were performed before and 4, 14, and 28 days post-injury and histological analyses of the TA muscle were performed at 14 days post-injury. Because I-R injury is endogenously recoverable in rodents [1], this group serves as a regenerative benchmark for successful healing. Animals were housed individually after surgery in standard caging and allowed food and water ad libitum. All animals were euthanized under isoflurane anesthesia with a lethal dose of pentobarbital (Fatal Plus). This study was conducted in compliance with the Animal Welfare Act, the implementing Animal Welfare Regulations and in accordance with the principles of the Guide for the Care and Use of Laboratory Animals. All animal procedures were approved by the US Army Institute of Surgical Research Institutional Animal Care and Use Committee (Protocol A-14-017).

### VML model

**Fig 1:**
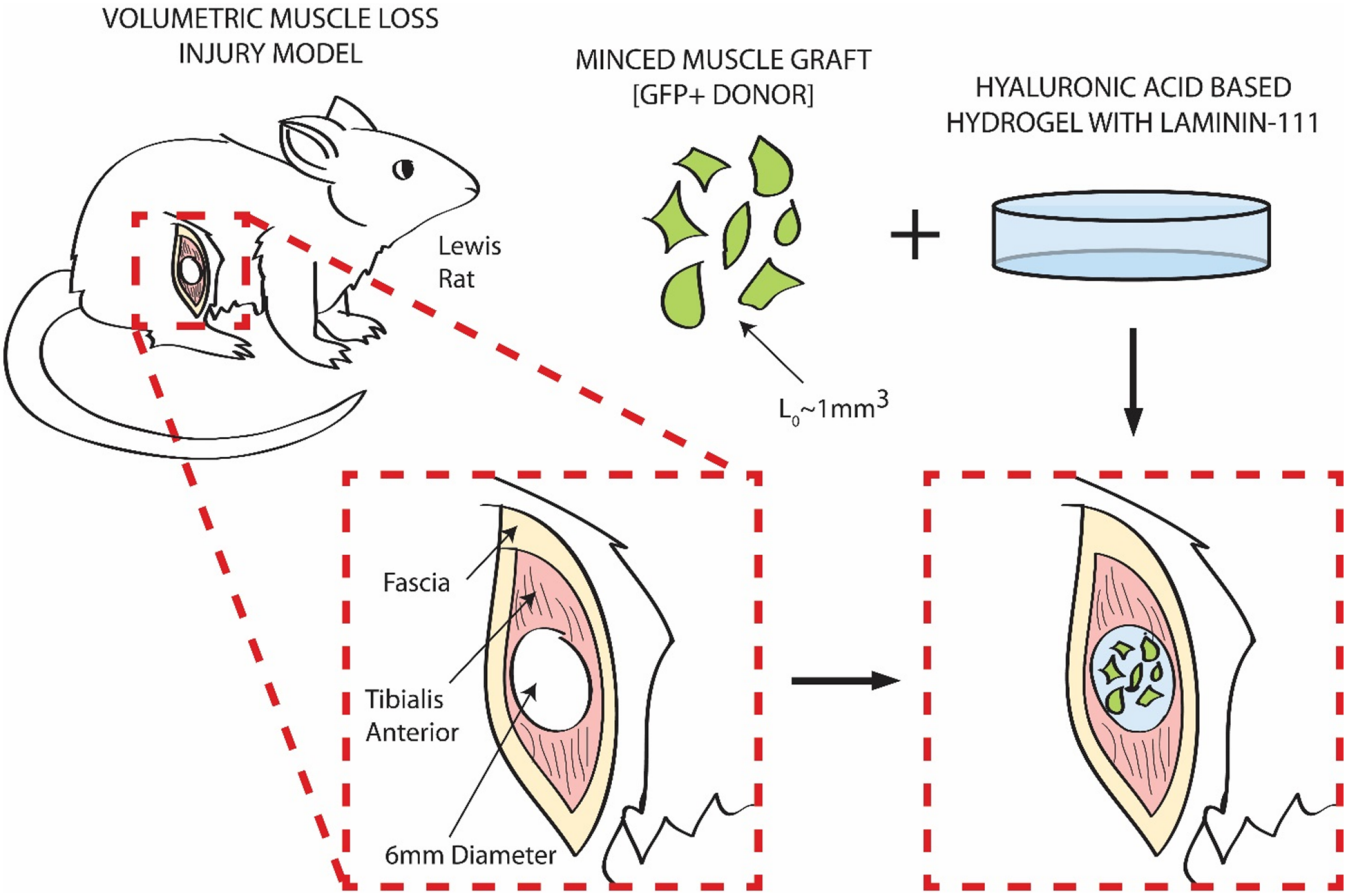
Rat model of volumetric muscle loss. The VML model consists of a 6mm full thickness surgical defect to the middle third of the TA muscle of male Lewis rats. The treatment paradigm requires the formation of a minced graft tissue construct ex-vivo utilizing a HA based hydrogel supplemented with laminin-111. The construct is then delivered to the defect site and the wound bed closed in layers. Minced graft material is procured from transgenic GFP^+^ Lewis rat donors.

Following previously reported procedures [8, 10], a lateral incision was made through the skin lengthwise along the lateral aspect of the tibialis anterior (TA) muscle of the left hind limb of adult male Lewis rats (Charles River Laboratories, Wilmington, MA, USA) with the animal under anesthesia (1–3% isoflurane) and in sterile conditions. After reflecting the skin and fascia from the anterior surface, a metal plate was inserted between the TA muscle and underlying extensor digitorum longus (EDL) muscle. A 6mm biopsy punch was used to excise a full thickness defect from the belly of the TA against the previously inserted metal plate. Wounds were either left unrepaired or repaired with therapies delivered directly to the VML defect region (Fig 1). The wound was closed in layers by suturing (fascia) and stapling (skin). Animals were administered buprenorphine SR approximately 30 minutes prior to surgery for post-operative pain relief and were monitored twice daily for the first three days following surgery to assess for complications with wound closure, dehydration, a pain management. No adverse events occurred following surgery.

### Ischemia-reperfusion injury

A subset of rats received an ischemia-reperfusion (I-R) injury to the left hind limb as a comparison group representing a fully recoverable skeletal muscle injury [1, 28, 29]. Briefly, with the animal under isoflurane anesthesia (1–3%) and in a supine position the left hind limb was exsanguinated by elevating it for 5 minutes prior to tourniquet application. A pneumatic digital tourniquet (Model DC1.6, D.E. Hokanson, Inc) attached to a PTS II Portable TK system (Delfi Medical Innovations, Inc) and air source (Model AG101) was placed as proximal as possible around the upper left limb. Ischemia was induced by inflating the cuff or 3 hours at 300 mmHg. Successful induction of I-R injury was confirmed by near complete ablation of muscle function 2–3 days after the procedure using *in vivo* functional assessment methodologies described below [1].

### Tissue construct preparation

Minced grafts were derived from the TA muscle of syngeneic GFP^+^ transgenic Lewis rats. The excised muscle tissue was minced into ~1mm^3^ pieces. After mincing, ~45mg of MG was mixed into a pre-polymer solution consisting of 1% w/v thiol-modified hyaluronan (Glycosil^®^, MW: 250 kDa, ESI BIO, Alameda, CA, USA), 1% PEGDA (Extralink^®^, MW: 3.4 kDa, ESI BIO, Alameda, CA, USA), and 50 μg/mL Laminin-111 (Cultrex^®^, Trevigen, Gaithersburg, MD, USA) in degassed sterile water. Hydrogel composition was selected based on an integration of results from multiple prior reports which optimized the base hydrogel system for cell viability and proliferation and the incorporation of laminin-111 into hydrogels for cell attachment [30–32]. For controls, constructs were simply prepared without MG and/or laminin-111 based on prescribed experimental group. The 45mg MG dose was chosen a priori to represent 50% of an approximately 90mg VML defect as determined based on prior studies utilizing the described method for producing a VML in the rat TA muscle [10, 13, 33–38]. The suspension was then cast and subsequently allowed to cross-link at 37°C for 30 minutes in a humidified incubator (5% CO_2_, 37°C).

### *In vivo* neuromuscular strength assessments

The animal was placed while under isoflurane gas anesthesia (see above) in a supine position with the knee and ankle joints fixed at right angles. The foot was strapped into a pedal coupled with a servomotor controlled force-displacement transducer (Aurora Scientific, Aurora, ON), and the peroneal nerve stimulated using percutaneous needle electrodes placed around the peroneal nerve. Optimal voltage was determined using a series of twitch and tetanic contractions. Contractile function of the TA muscle was assessed by first severing the distal tendon of the synergist EDL muscle and measuring peak isometric force as a function of stimulation frequency (400 ms train; 0.1 ms pulse width; 1–10 V; 10–200 Hz;) [9]. Force to torque conversion was performed using a standardized 3mm moment arm [39], which was then normalized to body weight.

### Histological & immunofluorescence analysis

A portion of the TA muscle from the defect region was embedded in a talcum-based gel, frozen in 2-methylbutane, and supercooled in liquid nitrogen. Cryosections (8 μm) were prepared and stained using standard protocols for hematoxylin & eosin. For immunofluorescence, sections were probed for laminin (1:100; catalog ab34360; Abcam), GFP (1:100; catalog ab6673; Abcam), CD68 (1:50; MCA341A488; BioRad), and Pax7 (5 μg/mL; AB_528428; Developmental Studies Hybridoma Bank) and detected with fluorescent secondary antibodies (1:200; catalog A11055, A21207, and A32723; Invitrogen). Brightfield and fluorescent images were acquired with a Zeiss Axio Scan.Z1 and stitched into a composite image depicting the cross section of the TA muscle belly. Qualitative assessments of morphology and composition were made by observing three sections from 5 muscles per group. Quantitative analyses were performed on the indexed image values after global thresholding and segmentation in MATLAB (Mathworks, Natick, MA, USA). For regional analyses, segmented images where multiplied with a binary mask representing each of the defect region and the remaining musculature.

### Statistical analysis

Data is reported as the mean ± SEM with statistically significant differences defined as p < 0.05 using analysis of variance with Tukey post-hoc tests for multiple comparisons. Sample sizes for quantitative immunohistochemistry and *in vivo* neuromuscular strength assessments are n = 3–5, and n = 6–8 per group and time point respectively.

## Results

### Gross anatomy

There were no differences between experimental groups with respect to body weight at either 2- or 8-weeks post-injury (Table 1). The mean wet weight of the VML defect was likewise similar across all groups, confirming that the surgical creation of the defect was highly reproducible. The wet weight of the EDL and TA muscles from the VML-injured limbs did not vary significantly between groups at either of the study endpoints, and TA wet weight was significantly lower for the affected limb relative to the unaffected contralateral limbs for all experimental groups at 8 Weeks.

**Table 1:** Gross anatomy and muscle weights.

Experimental Group	Duration	Defect Weight [mg]	Endpoint Body Weight [g]	TA Weight [mg]	EDL Weight [mg]
**No Repair**	2 Weeks	97.7 ± 5.5	404.5 ± 3.9	617.0 ± 14.3	154.3 ± 10.6
	8 Weeks	94.7 ± 4.1	456.3 ± 4.6	703.2 ± 17.6	216.2 ± 11.0
**HA**^†^	2 Weeks	84.1 ± 4.8	374.8 ± 7.1	610.6 ± 56.3	147.8 ± 11.0
	8 Weeks	90.1 ± 4.7	427.6 ± 3.3	702.7 ± 36.3	183.7 ± 9.6
**HA+LMN**	2 Weeks	91.8 ± 7.1	377.3 ± 6.3	622.0 ± 69.0	168.0 ± 5.1
	8 Weeks	90.8 ± 3.8	441.0 ± 5.9	681.3 ± 32.3	172.2 ± 14.2
**50% MG + HA**	2 Weeks	88.9 ± 6.0	373.2 ± 4.4	630.8 ± 61.5	143.4 ± 11.1
	8 Weeks	96.7 ± 4.7	460.0 ± 7.3	695.1 ± 57.5	200.1 ± 7.5
**50% MG + HA+LMN**	2 Weeks	85.0 ± 3.8	375.4 ± 5.2	653.4 ± 21.3	177.8 ± 3.1
	8 Weeks	90.3 ± 2.9	442.6 ± 6.9	706.7 ± 27.0	192.3 ± 11.9
**Contralateral Limbs**	2 Weeks	-	-	730.2 ± 5.9	167.6 ± 1.8
	8 Weeks			826.5 ± 8.9	190.1 ± 4.1

*Note*: Values are Mean ± SEM,

^†^Indicates subset of data from experimental group published previously [19].

### *In vivo* neuromuscular function assessments

**Fig 2:**
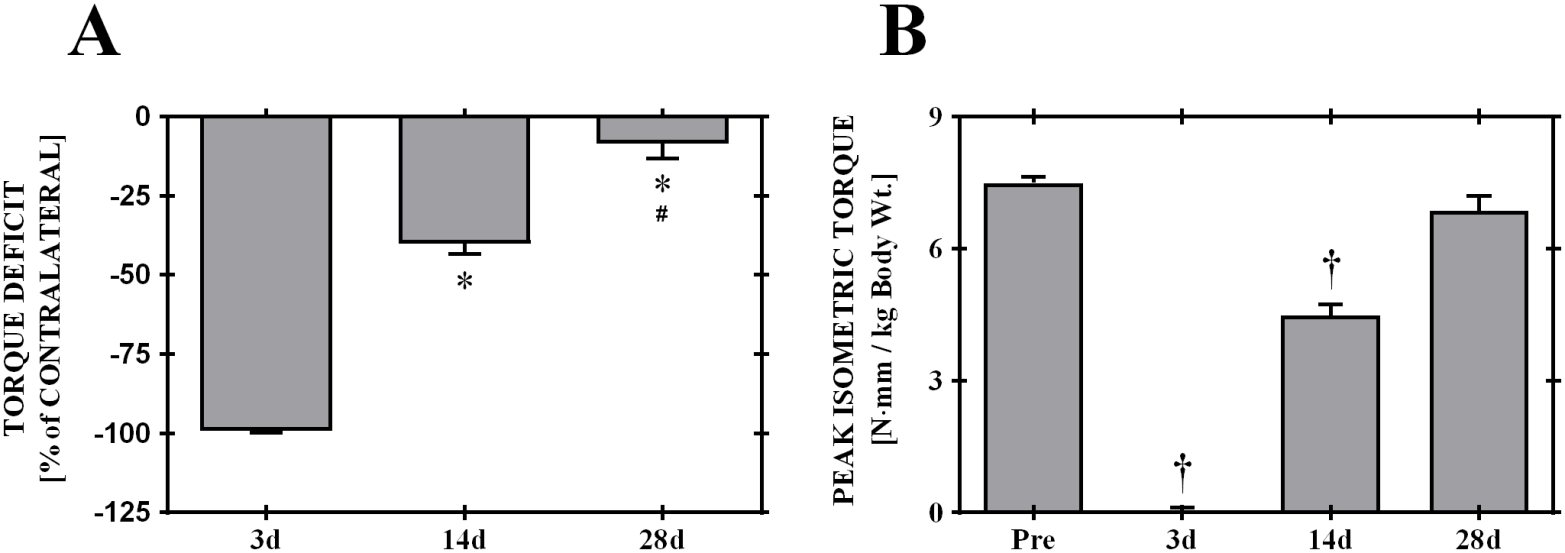
Functional recovery of ischemia-reperfusion injury. Peak isometric tetanic torques as a percentage deficit relative to pre-injury values (Panel A, left) and normalized to body weight (Panel B, right) show complete recovery of neuromuscular strength by 28 Days post-injury. * ≠ 3d, † ≠ 14d; p < 0.05. Values are means ± sem.

I-R injury was induced in a subset of rats to establish a benchmark for indices of successful skeletal muscle regeneration. I-R injury resulted in a 99.2 ± 0.6% strength deficit three days after injury, which recovered to -40.1 ± 3.2%, and -8.6 ± 4.6% at 14 and 28 days post-injury under unassisted endogenous mechanisms (Fig 2A). Isometric torque production 28 days post-injury was similar to pre-injury values (Pre- vs. 28 days: 7.5 ± 0.1 vs. 6.9 ± 0.3 N·mm; p = 0.467; Fig 2B).

**Fig 3:**
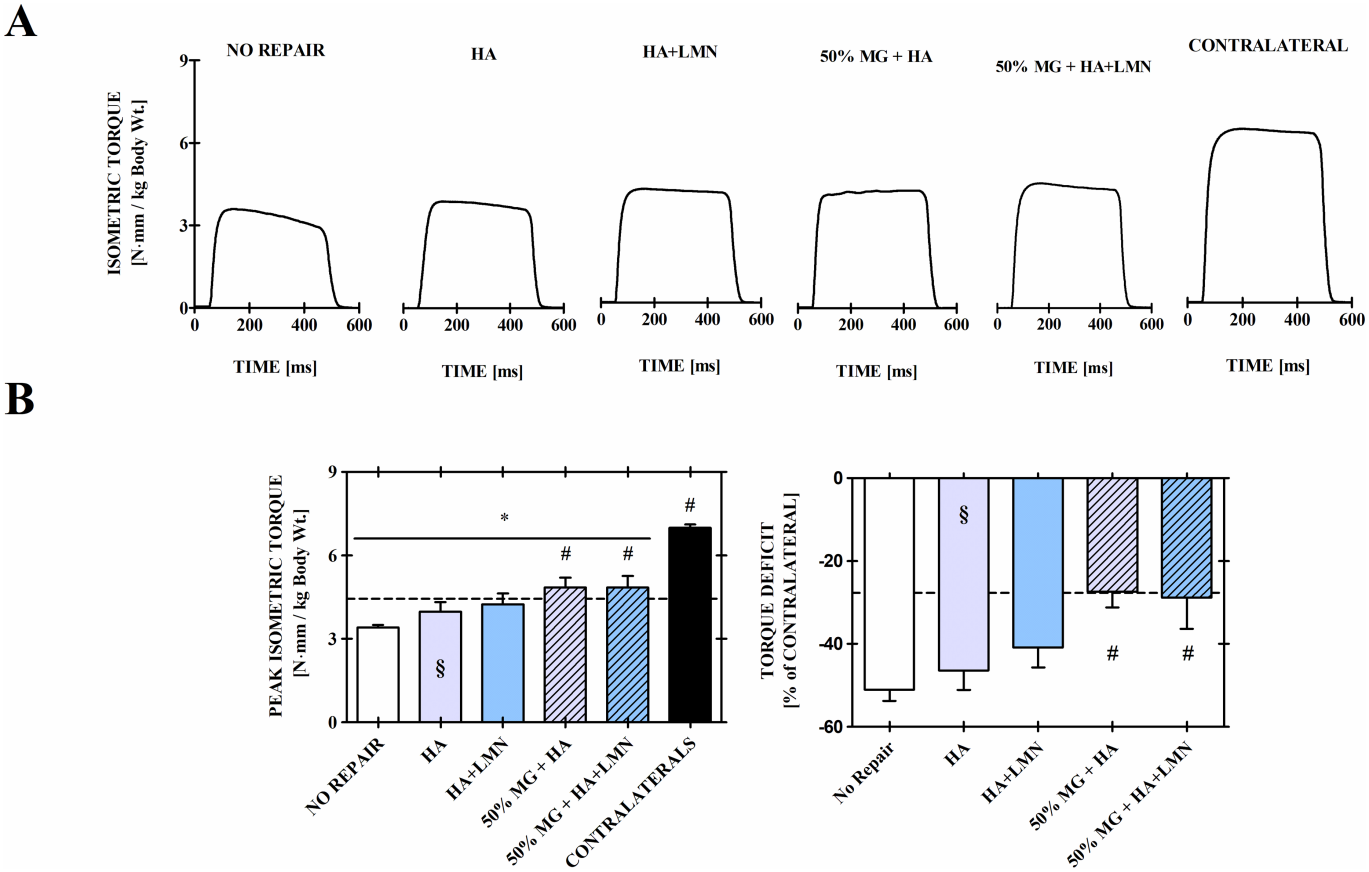
In vivo neuromuscular functional capacity is improved 8 weeks after repair with minced grafts delivered in a HA+LMN hydrogel. Physiological tetanic contraction waveforms (Panel A), peak isometric tetanic torque normalized to body weight at time of assessment (Panel B, left), and percent functional deficit relative to the matched contralateral limb (Panel B, right) show improved functional capacity in the 50% MG + HA+LMN group relative to the No Repair group. *Indicates a significant difference between specified groups and unaffected contralaterals, and ^#^indicates a significant difference between the specified group and no repair VML injury group; p < 0.05. Values are means ± sem. § Indicates set data for experimental group performed concurrently but published elsewhere [19]. Dashed reference lines represent mean values of a 50% MG control group from a concurrent study published elsewhere [13].

At 8-weeks post-VML injury, affected and contralateral limbs from all groups produced physiological tetanic contractions (Fig 3A) when stimulated at 150 Hz. Mean peak isometric torque production of unrepaired, VML affected limbs was 3.42 ± 0.09 N·mm/kg of body weight, representing a 51.0 ± 2.7% torque deficit compared with matched contralateral uninjured muscles (Fig 3B & 3C). Notably, at 8 weeks post-VML injury peak torque production remained significantly reduced compared to I-R injured muscles at 28 days post-injury (p < 0.0001), indicating the irrecoverable nature of traumatic VML injury. HA and HA+LMN treatment of the VML resulted in mean peak isometric torque production of 3.98 ± 0.34 N·mm/kg body weight (46.4 ± 4.7% deficit to matched contralateral limbs) and 4.24 ± 0.39 N·mm/kg body weight (40.9 ± 4.8% deficit to matched contralateral limbs), respectively. These values were not found to be significantly different from each other (p = 0.62) or the no repair group (p = 0.17, p = 0.08; respectively). Encapsulation of minced graft into either the base (HA) or HA+LMN scaffolding material, however, did result in a significant improvement of muscle function over the no repair group (p<0.05). Mean peak isometric torque production was 4.86 ± 0.34 N·mm/kg body weight (27.4 ± 3.8% deficit relative to matched contralateral limbs) and 4.84 ± 0.42 N·mm/kg body weight (28.9 ± 7.5% relative to matched contralateral limbs) for the 50% MG + HA and 50% MG + HA+LMN treatment groups, respectively. Notably, neither 50% MG + HA nor 50% MG + HA+LMN treatment groups significantly deviated from a recently reported 50% MG-only control group (4.47 ± 0.41; p = 0.49, p = 0.55) from our group [13]. Mean torque production of the unaffected contralateral limbs was 6.99 ± 0.12 N·mm/kg and did not vary significantly among groups, and all VML affected limbs regardless of treatment modality were significantly reduced compared with their unaffected contralateral limbs.

### Histological analysis

**Fig 4:**
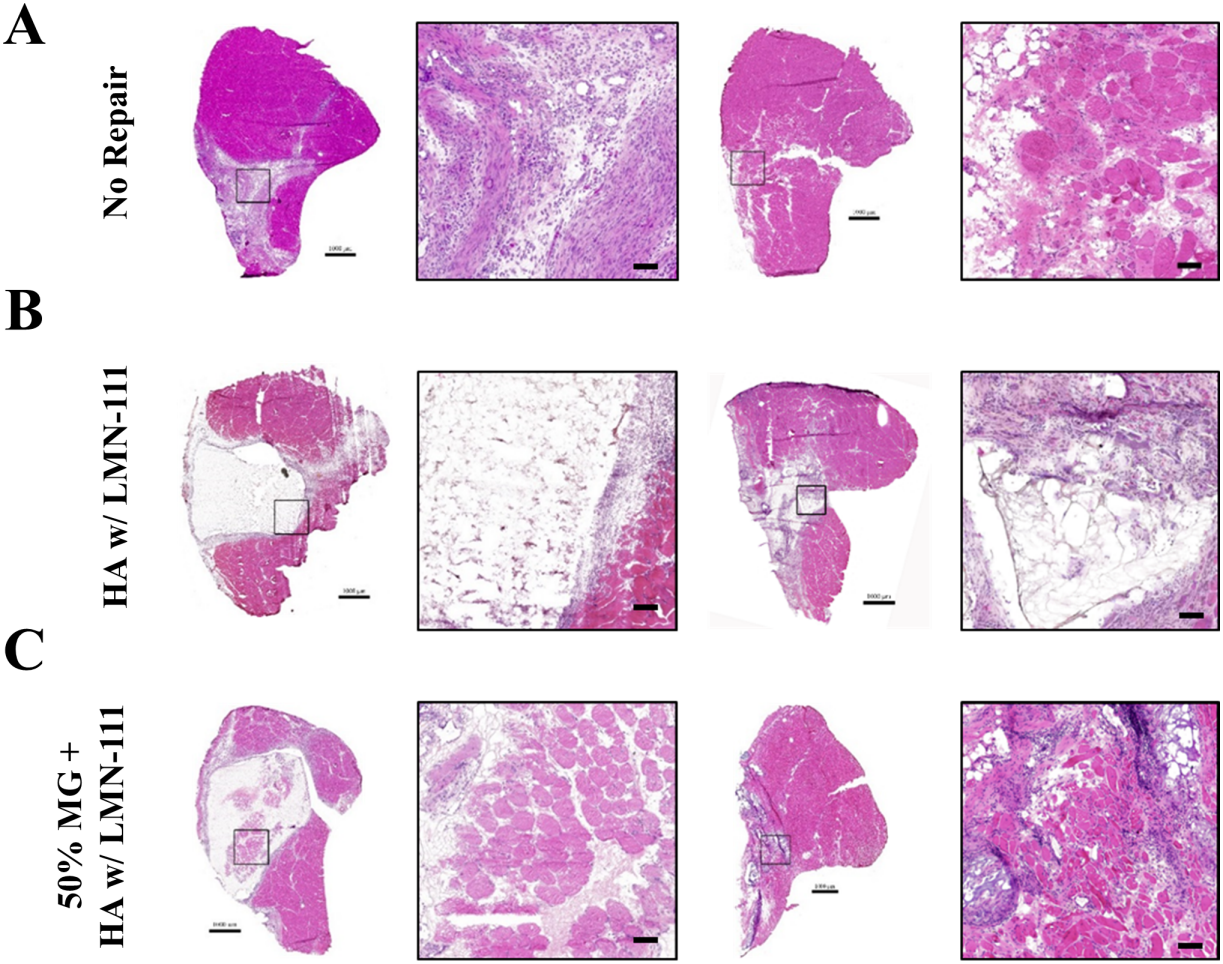
TA muscle histological analysis. Hematoxylin & Eosin staining of unrepaired VML affected limbs (top row), and VML affected limbs repaired with HA+LMN both with (bottom row) and without (middle row) minced muscle grafts at a dose of 50% of tissue mass deficit. Scale bars are 1mm for whole mount images, 50 μm for regions of interest.

**Fig 5:**
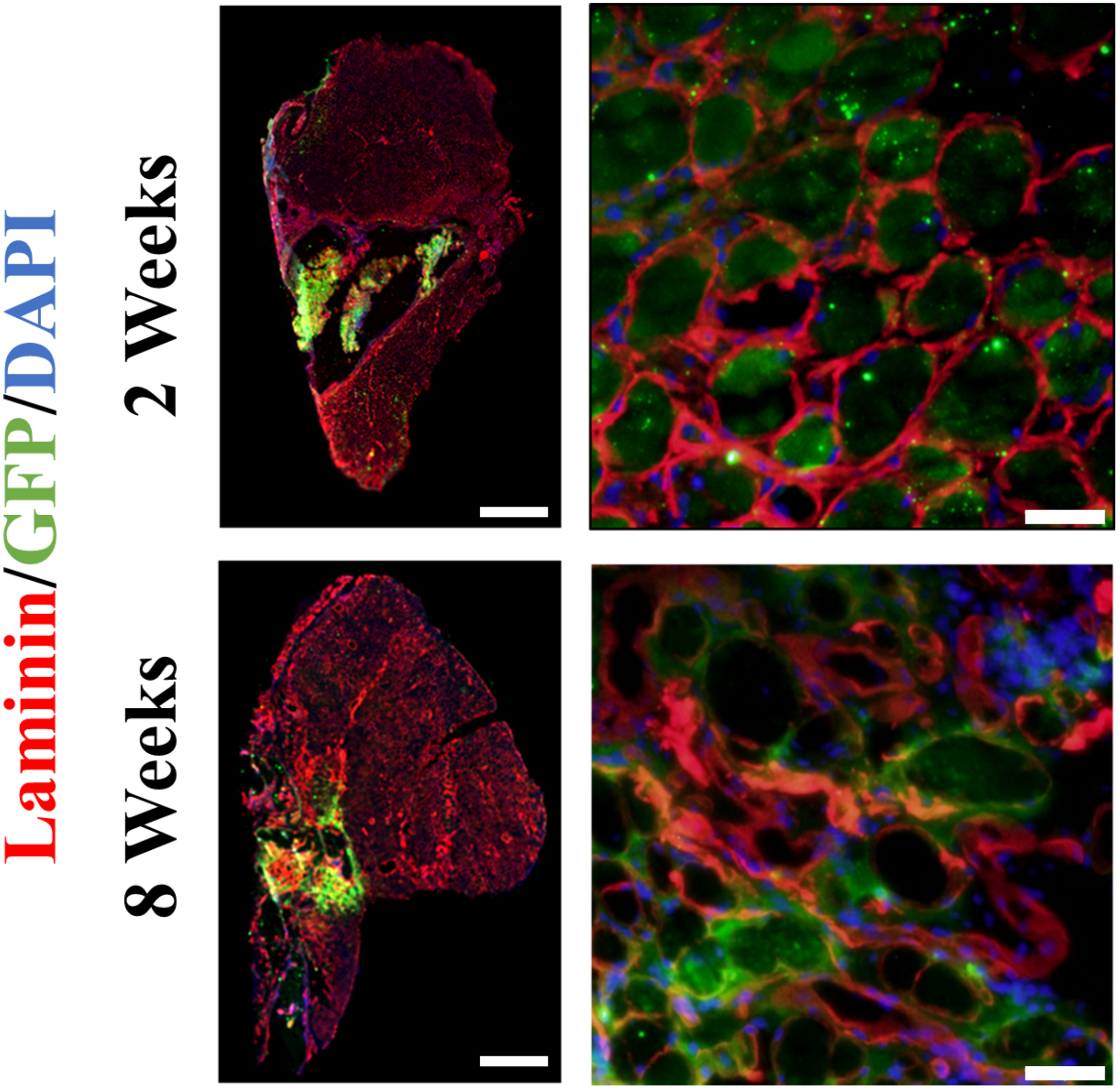
Immunofluorescence for assessment of GFP^+^ myofibers. VML affected muscle from the 50% MG + HA+LMN group were probed for the presence of GFP. GFP+ fibers were detected in a qualitatively similar magnitude at both 2 and 8 weeks post-injury indicating viable engraftment of donor derived muscle progenitor cells. Scale bars are 1mm for whole mount images, 50 μm for regions of interest.

Histologically, the VML-injured no repair group exhibited robust cellular infiltration of the defect space at 2 weeks post-injury that resulted in a mixed healing outcome at 8 weeks consisting of myofibers, which sparsely populate a region predominately filled by extracellular matrix deposition (Fig 4A). The HA+LMN group showed minimal cellular infiltration of the defect region at 2 weeks owing to the continued presence of the largely intact scaffolding. By 8 weeks, the scaffolding was largely degraded, and the portions that remained intact within the defect space where surrounded by cellular infiltrate and associated matrix deposition (Fig 4B). For the 50% MG + HA+LMN repair groups, regenerating myofibers were present at 2 weeks post injury, and appeared to be of similar diameter to healthy fibers from the unaffected regions of the musculature (Fig 4C). By 8 weeks, the scaffolding was observed to be largely degraded, and the defect space filled with a mixture of regenerated myofibers and matrix deposition. GFP was detected in the defect region of the 50% MG + HA+LMN treated muscles at both 2 and 8 weeks post-injury indicating survival of the graft derived progenitor cells and their involvement in the myofiber regeneration (Fig 5). Qualitative observation of GFP^+^ staining indicated that the majority of the regenerated myofibers within the defect region were minced graft derived, as observed previously with minced graft-only repair [13, 15, 40].

**Fig 6:**
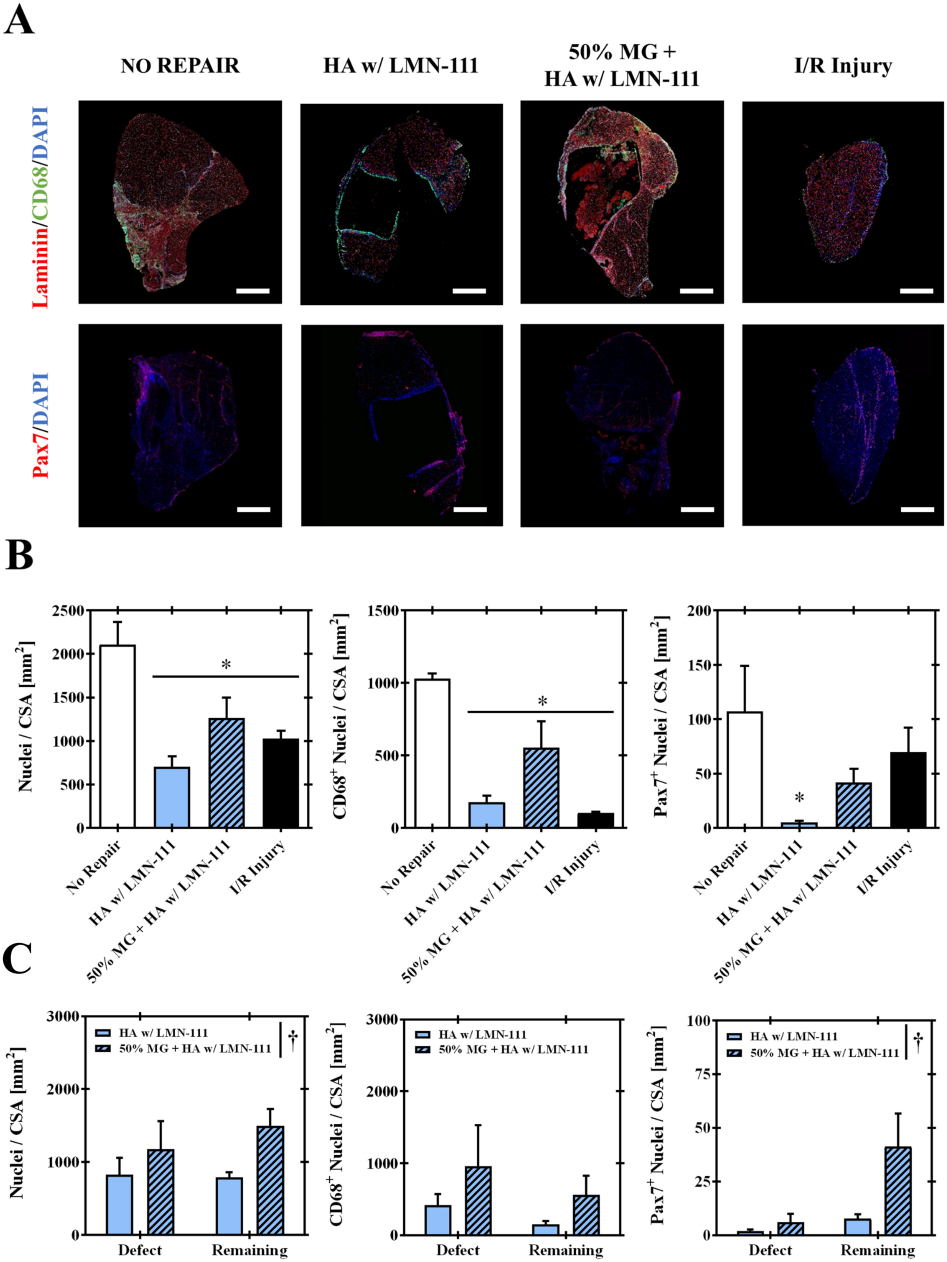
Quantitative immunofluorescence for assessment of cellular composition. Two weeks post-injury VML affected and I-R injured muscles were separately probed for Pax7 and CD68 to assess locality and density of satellite cell and macrophage populations. A) Composite images of the muscle belly cross-sections were capture and digitally enumerated. B) Whole muscle and C) regional analysis of specified cellular infiltration were performed. *Indicates significant difference (p<0.05) relative to No Repair group. ^†^Indicates significant main effect of group (p<0.05) by 2-way ANOVA of the specified independent variable. Values are means ± sem. Scale bars are 1 mm.

Quantitative analysis of the early wound healing response two weeks post-injury indicated that whole muscle cellularity (i.e. nuclei counts, DAPI) was significantly greater (p<0.05) in the non-repaired VML group relative to both HA+LMN based treatment groups and limbs injured via ischemia-reperfusion (Fig 6). Similarly, CD68^+^ cells were attenuated in repaired groups compared with non-repaired muscles, and presented values similar to muscle two weeks after I-R injury. Pax7^+^ cell number was attenuated in the HA+LMN group, likely owing to the presence of a largely undegraded scaffolding. The inclusion of minced grafts in the scaffolding (i.e. 50% MG+ HA+LMN) accordingly produced a nominal increase in pax7^+^ cells such that the cellular density approximated the values of the positive healing control group (i.e. I-R Injury).

Sub-regional analysis of the defect region and remaining muscle mass were performed to delineate any differences between VML-repaired groups. Gross cellularity was significantly increased regardless of region (ANOVA; Interaction p = 0.480, Group p = 0.043, Region p 0.570), which was partly attributed to an increased proportion of Pax7^+^ cells throughout the muscle (ANOVA; Interaction p = 0.058, Group p = 0.017, Region p = 0.090). CD68^+^ cell number was not statistically different between groups in either muscle region (ANOVA; Interaction p = 0.817, Group p = 0.118, Region p = 0.259).

## Discussion

In this study, delivery of a minced muscle graft in a dose of 50% of the VML defect mass within a laminin-111 supplemented, hyaluronic acid based hydrogel material resulted in the improvement of *in vivo* functional capacity of a VML affected limb. Specifically, this combination therapy increased peak tetanic torque production by 42% over an unrepaired VML injury. This observation is in line with previous reports demonstrating strength gains with a reduced dose of minced muscle grafts co-delivered with both collagen [40] and acellular bioscaffolds at 8- and 12-weeks post-injury [12, 13]. This response, however, was not found to be significantly different from either the 50% MG + HA group or our previously published benchmark for the delivery of 50% MG without accompanying scaffolding [13] suggesting neither the HA in isolation or the in combination with laminin-111 contributed significantly to whole muscle function beyond the effects of the supplied MG material. Moreover, while graft-mediated *de novo* muscle fiber regeneration was indicated by the presence of GFP^+^ muscle fibers, as demonstrated previously [13, 15, 40], the robustness of the regenerative response was qualitatively in line with a recent observation of tissue equivalent (50% MG-only) implantation and was clearly lesser than a 100% MG repair [13].

As envisaged, an optimal MG expansion scaffolding would provide a substrate for graft and host-derived satellite cells and other myogenic progenitors to initiate myotube and eventual myofiber formation within the otherwise non-regenerative defect region. The net effect would be a greater number of regenerated muscle fibers than accounted for by the implantation of an equivalent amount of minced graft alone. Prior reports using autologous devitalized minced muscle grafts, collagen hydrogel, or an acellular bioscaffold co-delivered with vital minced grafts either did not observe noteworthy augmentation of the graft, indicating that each material temporarily served simply as a volumetric extender until eventual degradation [8, 16, 41] or had a deleterious effect on graft mediated regeneration [13]. The results of this study align most closely with the conclusion that the HA+LMN does not augment the MG mediated regenerative response in a significant way as the scaffolding material did not suppress the viability or engraftment of graft derived progenitor cells nor was it sufficient in promoting a robust graft mediated regenerative response as intended.

In light of this suboptimal outcome, further analysis was performed to identify potential mechanisms by which graft mediated regeneration failed to thrive when co-delivered with the HA+LMN scaffolding evaluated herein so as to inform future efforts at optimizing this approach. Of particular interest to these analyses were the relative population densities of satellite cells and macrophages within the affected musculature given their importance in mediating skeletal muscle regeneration.

Focusing first on the macrophage response, the HA+LMN based scaffolds and tissue constructs (i.e. 50% MG + HA+LMN) did appear to have a damping effect on the local macrophage response to VML. It is well established that in addition to satellite cell activation [42], adequate revascularization [43], neural support [44], and appropriate inflammatory response are critical to skeletal muscle regeneration [45]. Specifically, VML promotes a dysregulated inflammatory response [46, 47], demonstrated herein as a heightened macrophage presence 14 days post-injury relative to a canonically regenerating I-R injury. The observation that HA+LMN based treatments attenuate the seemingly pathological macrophage response to VML injury supports prior observations indicating that myeloid and T-lymphocyte immune responses were modulated by decellularized extracellular matrix implantation in a mouse quadriceps VML model [47]. Both the prior and current study associate attenuated inflammation with improved functional recovery, but little evidence of *de novo* muscle fiber regeneration when implanting the biomaterial alone. Conversely, prior reports have also demonstrated that biomaterial implantation (e.g., commercially available decellularized small intestine sub-mucosa [48] and commercially available micronized decellularized urinary bladder matrix [13]) exacerbates the already heightened immune response to VML injury and effectively suppresses regenerative outcomes. Together, these findings signify that biomaterial augmentation of the VML injury-induced immune response is not, in and of itself, sufficient to orchestrate *de novo* muscle fiber regeneration, and biomaterial-related exacerbation of the immune response can have deleterious effects on VML wound healing.

Given that the macrophage response to the HA+LMN based scaffold and tissue construct treatments more closely aligned with the positive healing control (i.e. I-R Injury) than with the pathological unrepaired VML condition, dysregulated satellite cell dynamics were hypothesized to be the predominate limitation to the observed regenerative response. The primary therapeutic focus regarding tissue regeneration in this study and most studies in the field is placed on the VML defect, and therefore it is intriguing that the 50% MG + HA+LMN treatment augmented cellular responses specifically within the remaining musculature as both total nuclei and Pax7^+^ cells were heightened in the remaining musculature compared to biomaterial implantation-alone. Notably, the elevation of Pax7^+^ cells throughout the muscle served to normalize satellite cell content to values observed in a time-matched canonical regenerative response following I-R injury. Given the apparent low regenerative response within the VML defect, it is likely that the improved functional recovery following implantation of minced muscle grafts with co-delivered with HA+LMN is due to a superposition of *de novo* muscle fiber regeneration and augmented repair of muscle fibers within the remaining musculature. As such, two key takeaways from these observations are: (1) that significant migration of host satellite cells from the remaining muscle mass remains a primary limitation to *de novo* muscle fiber regeneration within a VML defect [49]; and (2) given the abundance of remaining musculature that functions sub-optimally after VML injury [18], the capacity of implanted biomaterials to ameliorate regeneration within the remaining musculature may be of profound clinical benefit.

## Conclusions

This study was initially motivated by the imbalanced relationship between the supply and demand of autologous donor tissue required for the treatment of large VML defects and multiply injured patients, and the findings presented herein bring forth a number of important considerations regarding reduced dose minced muscle graft therapies to VML. Based on the results of this study and others, it is clear that minced graft therapies at doses lower than the magnitude of tissue loss can drive functional improvements in neuromuscular strength in the presence of a biomaterial scaffolding [7, 12]. The limitation of this approach, however, is that *in vivo* expansion of this graft-derived material is not yet optimized. The results of this study reemphasize the importance of reinstituting an appropriate inflammatory response as a key component of regenerative strategies to VML injury, but highlight the necessity of satellite cell inclusion to interact with appropriate immune responses to achieve *de novo* muscle fiber regeneration. Lastly, the current work highlights the potential for VML therapies to augment regeneration and repair within the remaining musculature, a finding that may carry currently significant clinical impact.
